# Acute exercise remodels mitochondrial membrane interactions in mouse skeletal muscle

**DOI:** 10.1152/japplphysiol.00819.2013

**Published:** 2013-08-22

**Authors:** Martin Picard, Benoit J. Gentil, Meagan J. McManus, Kathryn White, Kyle St. Louis, Sarah E. Gartside, Douglas C. Wallace, Douglass M. Turnbull

**Affiliations:** ^1^Mitochondrial Research Group, Institute for Ageing and Health, University of Newcastle, Newcastle upon Tyne, United Kingdom;; ^2^Center for Mitochondrial and Epigenomic Medicine, The Children's Hospital of Philadelphia and University of Pennsylvania, Department of Laboratory Medicine, Philadelphia, Pennsylvania;; ^3^Department of Neurology/Neurosurgery and Montreal Neurological Institute, McGill University, Montreal, Quebec, Canada;; ^4^EM Research Services, University of Newcastle, Newcastle upon Tyne, United Kingdom;; ^5^Institute of Neuroscience, University of Newcastle, Newcastle upon Tyne, United Kingdom;; ^6^Newcastle University Centre for Brain Ageing and Vitality, Institute for Ageing and Health, University of Newcastle, Newcastle upon Tyne, United Kingdom; and; ^7^Wellcome Trust Centre for Mitochondrial Research, Institute for Ageing and Health, University of Newcastle, Newcastle upon Tyne, United Kingdom

**Keywords:** mitochondrial dynamics, skeletal muscle, exercise, fusion, metabolic state

## Abstract

A unique property of mitochondria in mammalian cells is their ability to physically interact and undergo dynamic events of fusion/fission that remodel their morphology and possibly their function. In cultured cells, metabolic perturbations similar to those incurred during exercise influence mitochondrial fusion and fission processes, but it is unknown whether exercise acutely alters mitochondrial morphology and/or membrane interactions in vivo. To study this question, we subjected mice to a 3-h voluntarily exercise intervention following their normal physical activity patterns, and quantified mitochondrial morphology and membrane interactions in the soleus using a quantitative electron microscopy approach. A single exercise bout effectively decreased blood glucose (*P* < 0.05) and intramyocellular lipid content (*P* < 0.01), indicating increased muscle metabolic demand. The number of mitochondria spanning Z-lines and proportion of electron-dense contact sites (EDCS) between adjacent mitochondrial membranes were increased immediately after exercise among both subsarcolemmal (+116%, *P* < 0.05) and intermyofibrillar mitochondria (+191%, *P* < 0.001), indicating increased physical interactions. Mitochondrial morphology, and abundance of the mitochondrial pro-fusion proteins Mfn2 and OPA1 were unchanged. Collectively, these results support the notion that mitochondrial membrane dynamics are actively remodelled in skeletal muscle, which may be regulated by contractile activity and the metabolic state. Future studies are required to understand the implications of mitochondrial dynamics in skeletal muscle physiology during exercise and inactivity.

in response to contraction, skeletal muscle fibers undergo significant metabolic and molecular remodelling contributing to the health benefits of exercise (14). These changes include but are not limited to translocation of glucose transporters (GLUT4) to the plasma membrane (32), allosteric activation of Ca^2+^-sensitive mitochondrial dehydogenases (36), demethylation of mitochondrial gene promoters in the nuclear genome (3), and phosphorylation of several transcription factors (25). Collectively, these acute (minutes to hours) changes enable adequate adaptation in the face of rising energy demands. Most of skeletal muscle energy requirements during submaximal exercise are met by mitochondria, specialized organelles that exhibit unique topology and morphology within myofibers (2, 29, 37, 43). An emerging aspect of mitochondrial biology is their ability to undergo dynamic morphological changes (6), which enable quality-control processes (51), preserve mitochondrial DNA integrity (9), and possibly modulate mitochondrial respiratory capacity, reactive oxygen species (ROS) production, and sensitivity to permeability transition (7, 40).

Mitochondrial morphology transitions in isolated cells occur in response to variations in cellular energy metabolism (34), whereby energetic deprivation (metabolic *under*supply) acutely favours mitochondrial elongation (22, 46) and excess substrate supply (metabolic *over*supply) lead to mitochondrial fragmentation (50, 53) (see Ref. 42 for a discussion). The redox state and oxidative stress can also modulate mitochondrial morphology (15, 47). Furthermore, we recently demonstrated the existence of membrane interactions among skeletal muscle mitochondria in vivo (43), and others have observed adjacent mitochondria exchanging matrix-located fluorescent molecules in myofibers (39) and cardiomyocytes (26), which constitute evidence of mitochondrial dynamics. However, whether an acute bout of exercise can remodel mitochondrial membrane interactions or acutely alter mitochondrial morphology is unknown.

To study this question, key technical and physiological factors were considered. First, examining skeletal muscle immediately following *exhaustive* exercise may cause mitochondria swelling and cristolysis (18, 21) rendering impracticable the study of subtle changes in mitochondrial morphology. Second, given that gene expression and hormonal mediators regulating cellular energy metabolism are under strong diurnal control (1, 17, 28), the timing of exercise interventions with the natural physical activity patterns of nocturnal laboratory animals appears essential to isolate the effect of physical activity. Third, psychological stress that may impinge upon animals during forced running or swimming paradigms could possibly have confounding deleterious effects on mitochondrial morphology and function (23, 35). Finally, a quantitative high-resolution approach is required to resolve mitochondrial membrane interactions and compare key aspects of mitochondrial morphology (43).

To meet these requirements, we established a nonstressful voluntary exercise paradigm by introducing a running wheel at the onset of the dark phase in the light-dark cycle, and examined mitochondrial morphology and membrane interactions immediately after exercise by using a novel electron microscopy approach combining views from two orthogonal planes (43). Furthermore, we applied our analyses to the two major mitochondrial populations in myofibers: subsarcolemmal (SS) and intermyofibrillar (IMF), which exhibit both functional (10, 31) and morphological (20, 43) differences. We complemented these measurements with assessment of key proteins known to mediate mitochondrial membrane interactions. Overall, our findings demonstrate that a single exercise bout acutely remodels mitochondrial membrane interactions, but not morphology, in mouse skeletal muscle.

## METHODS

### 

#### Animals and experimental design.

**Fig. 1.**
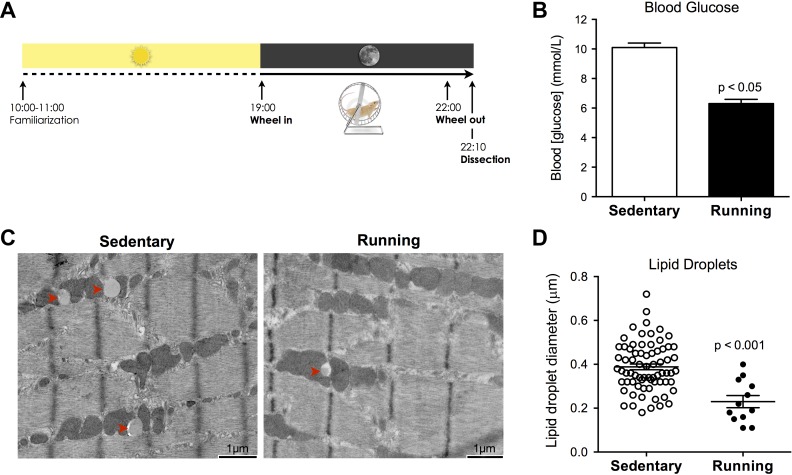
A single bout of exercise modulates skeletal muscle metabolic state. *A*: experimental paradigm showing the period (1 h) of familiarization and acclimatization early during the light phase, followed by a single 3-h period with free access to a running wheel during the dark phase of the light/dark cycle. *B*: blood glucose levels measured immediately after exercise (means ± SE). *C*: electron micrographs of the soleus depicting intramyocellular lipid droplets from sedentary and running exercised animals. *D*: quantification of lipid droplet size and number matched for surface area of muscle analyzed. Bars are means ± SE; *n* = 15–16 myofibers analyzed per group.

Eight-week-old female C57BL/6J mice were group housed with free access to food and water and maintained at constant temperature on a 12:12-h light-dark cycle. At 1000 on the day of experiments, a pair of mice derived from the same litter were randomly assigned to “sedentary” or “running” conditions, and transferred individually to new cages with normal access to food and water, layered with ∼3 cm of wood-chip bedding, and containing 1 cup of paper-shredded nestlet in a cage corner. For mice assigned to the running condition, the cage contained a 15-cm diameter plastic running wheel equipped with a magnetic rotation counter, whereas for the sedentary mice only the wheel support was introduced to control for the presence of a novel object. At 1100, the running wheel and support were removed from the cages, and mice were kept in a quiet room thereafter. Immediately before the onset of the dark phase of the light cycle (1855), the running wheel or wheel support were reintroduced, such that mice had undisrupted access to the wheel at 1900 when the lights were turned off. For the remaining 3 h, mice remained undisturbed in the dark. The experimental paradigm is illustrated in Fig. 1*A*.

At 2200, lights were turned on, at which point mice stopped running. At 2210, mice in the running condition were killed by cervical dislocation (2215 for the sedentary mice). Blood glucose was measured immediately from trunk blood using a glucose monitor (AlphaTrak, Abbott). Within 1 to 1.5 min following decapitation, the soleus was dissected, cut into two equal halves, and fixed by immersion into fixative for transmission [transmission electron microscopy (TEM)] and scanning electron microscopy (SEM). Tissues remained in fixative overnight at room temperature, and >48 h at 4°C before processing for electron microscopy. Running distance was monitored by the number of wheel revolution over the 3-h period. Experiments were repeated three times, and a total of six mice were used. All animal procedures were approved by the University of Newcastle Animal Care and Ethics Committee (ACEC).

#### Transmission electron microscopy.

For TEM, half of the mouse soleus was immediately fixed in a 2% glutaraldehyde solution in 0.1 M cacodylate (TAAB Lab Equipment) buffer, pH 7.4 as described previously (43). Briefly, muscle samples were then postfixed and dehydrated, after which the half soleus was cut into smaller segments and embedded either in transverse (TS) or longitudinal (LS) orientation in 100% resin. Orientation and section quality was checked with 1-μm thick sections, and ultrathin sections of 70 nm were subsequently cut using a diamond knife on a Leica EM UC7 ultramicrotome. Sections were stretched with chloroform to eliminate compression and mounted on Pioloform filmed copper grids prior to staining with 2% aqueous uranyl acetate and lead citrate (Leica). Ultrathin sections were examined on a Phillips CM 100 Compustage (FEI) transmission electron microscope and digital micrographs were captured by an AMT CCD camera (Deben).

To analyze SS mitochondrial morphology and lipid droplets, muscle in the LS orientation was photographed at ×19,000 magnification. Mitochondria-rich regions were selected for analyses. Lipid droplets were quantified by measuring the diameter of all lipid droplets present in randomly selected micrographs in both groups. For IMF mitochondria, muscle in the TS orientation was imaged at ×7,900 magnification. To insure optimal transverse orientation at the subcellular level (relative to the Z-line), only muscle fibers presenting no more than two Z-lines separated by no less than 10 to 15 μm were selected for analysis (43). For each animal, five to six mitochondria-rich muscle fibers were analyzed in both TS and LS, for which at least two micrographs were captured, allowing the analysis of ∼40 IMF and 40 SS mitochondria per myofiber. A total of 505 (sedentary, SED) and 563 (running, RUN) SS, and 653 (SED) and 623 (RUN) IMF mitochondria were analyzed. Part of the data from the sedentary animals was previously reported in (43). For all morphological parameters, the coefficient of variation was significantly greater between different myofibers of a given animal than between animals.

To quantify mitochondrial interactions among SS and IMF mitochondria, micrographs of muscle photographed in the longitudinal orientation at 19,000 (SS) and 13,500 (IMF) were used. In the IMF compartment, a total of 750 (SED) and 636 (RUN) Z-lines possessing mitochondria on both sides were analyzed, either as pairs of discrete organelles, a continuous mitochondrion, or interacting organelles. The proportion of Z-lines spanned by a continuous mitochondrion or interacting mitochondria is reported as a percentage of the total. In addition, 437 (SED) and 391 (RUN) SS, and 667 (SED) and 489 (RUN) IMF physical contacts sites between adjacent mitochondria were evaluated for the presence of electron density contact sites (EDCS). The proportion of all mitochondrial contact sites between organelles that were electron dense is reported as a percentage of total contacts.

#### Scanning electron microscopy.

For SEM, the other half of the mouse soleus was immediately fixed in a 1% glutaraldehyde (TAAB Lab Equipment) and 0.5% paraformaldehyde (Sigma 158127) solution in a 0.060 M cacodylate (TAAB Lab Equipment) buffer, pH 7.4 as described previously (43). For processing, fixed samples were rinsed twice in 0.067 M cacodylate buffer and subsequently immersed in DMSO (Sigma D2650) for 5 to 10 min. Then, the fixed samples were snap frozen in liquid nitrogen-cooled isopentane (Sigma 277258) for 5 to 10 s and immediately fractured by applying lateral pressure within a liquid nitrogen-cooled cracking instrument. The resulting fractured specimens, ∼0.5 to 3 mm in size, were rinsed three times in 0.067 M cacodylate buffer and postfixed in osmium tetroxide (OsO_4_) 1% for 1 h. Samples were then transferred to OsO_4_ 0.1% for 72–96 h to partially extract cytoplasmic components (37). Then, muscle samples were dehydrated in sequential steps of ethanol (25%, 50%, 75%, and 100% twice) and dried with CO_2_ in a Baltec Critical Point Dryer. Specimens were mounted on stubs covered with carbon disks, gold coated (15 nm) in Polaron SEM Coating Unit, and examined on a Stereoscan 240 Scanning Electron Microscope. Fracture sites were observed for exposed mitochondria and digital micrographs were captured at different magnifications with the Orion 6.60.6 software.

#### Confocal microscopy.

**Fig. 2.**
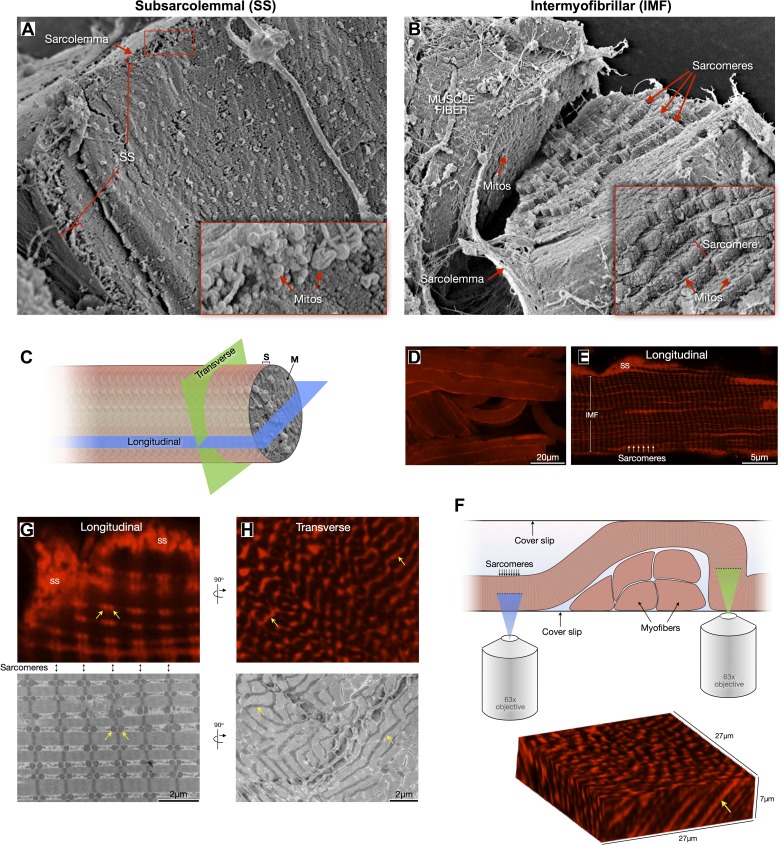
Topological and morphological differences between subsarcolemmal (SS) and intermyofibrillar (IMF) mitochondria. *A*: scanning electron microscopy (SEM) imaging of a freeze-fractured soleus muscle fiber sectioned in the cross-sectional plane with its cytoplasm exposed. The SS region is outlined where mostly globular SS mitochondria are clustered. *B*: SEM of freeze-fractured muscle fiber cracked to expose the staircase-like sarcomere structure revealing the IMF mitochondrial reticulum between each sarcomeric plane. *C*: diagram representing the two main planes—longitudinal and transverse (i.e., cross-section)—used to quantify various aspects of mitochondrial morphology. *D* and *E*: confocal imaging of permeabilized muscle fibers labeled with Mitotracker Red showing SS and IMF mitochondria and sarcomeric organization. *F*: schematic of experimental setting used for optical sectioning of Mitotracker-labeled muscle fibers in the longitudinal (blue) and cross-sectional (green) planes. Below is a three-dimensional reconstruction of an oblique section across a myofiber, showing elongated organelles (arrow). *G*: the longitudinal view in both confocal and electron microscopy reveals the pairwise arrangement of IMF mitochondria across Z-lines at each sarcomeric plane (appearing as spherical organelles, arrows), whereas the transverse view reveals the actual tubular morphology of IMF mitochondria (*H*, arrows).

To image mitochondria by confocal microscopy, permeabilized myofibers were prepared as described previously (41). Briefly, a fresh mouse soleus was finely dissected on ice in relaxing buffer, permeabilized with saponin 0.05 mg/ml for 30 min, rinsed 3 times, and incubated for 20 min in Mitotracker Red ROX (Life Technologies M7512) 15 μM at 30°C. Stained muscle fibers were then sandwiched between a glass bottom 12-well plate (MatTek, P12G-1.5–14F) and circular coverslip. Images were immediately acquired using an inverted laser scanning confocal microscope (Zeiss LSM 710) with a PlanApo 63x/1.40 oil immersion objective. In this setting, most fibers lie in perfect longitudinal orientation when imaged (see Fig. 2*D*), although some overlapping fibers produced perpendicular segments enabling cross-sectional (transverse) imaging (see Fig. 2*F*). Three-dimensional reconstructions of Z-stacks were produced by the Zen software using default settings.

#### Morphological analyses and statistics.

Mitochondrial shape descriptors and size measurements were obtained using Image J (version 1.42q, NIH, http://rsb.info.nih.gov/ij) by manually tracing only clearly discernible outlines of SS and IMF mitochondria on TEM micrographs, as described previously (43) and shown in Fig. 4*D*. Surface area (or mitochondrial size) is reported in μm^2^; perimeter in μm. Aspect ratio (AR) is computed as [(major axis)/(minor axis)] and reflects the “length to width ratio”; form factor (FF) [(perimeter^2^)/(4π·surface area)] reflects the complexity and branching aspect of mitochondria; circularity [4π·(surface area/perimeter^2^)] and roundness [4·(surface area)/(π·major axis^2^)] are two-dimensional indexes of sphericity with values of 1 indicating perfect spheroids; and Feret's diameter represents the longest distance (μm) between any two points within a given mitochondrion (30).

Computed values were imported into Microsoft Excel and Prism 6 (GraphPad Software) for data analysis. Statistical significance was evaluated based on 95% confidence interval (C.I.) of the mean. To produce frequency distributions of morphological parameters, each mitochondrion was assigned to one of twenty bins of equal sizes and proportions were determined to produce frequency histograms.

#### Western blot analyses.

Thirty micrograms of muscle homogenates from the extensor digitorum longus (EDL), the soleus (Sol), the red gastrocnemius (RGas), and the white gastrocnemius (WGas) were prepared with Laemmeli buffer in reducing conditions. Protein abundance was analysed by Western blot using antibodies against mitofusin 2 (Mfn2) (Sigma), optic atrophy 1 (OPA1) (BD biosciences), translocase of outer mitochondrial membrane 20 kDa (TOM20) (Santa Cruz Biotech) and GAPDH (Abcam). Band intensities from immunoblots were quantified using ImageJ from three mice per condition, and normalized against GAPDH.

## RESULTS

### 

#### Exercise and the metabolic state.

During a 3-h period with free access to running wheel during the early part of the dark cycle (Fig. 1*A*), exercising mice ran intermittently for a total distance of 1893 ± 150 (mean ± SD) meters. To assess whether this single acute bout of exercise altered the metabolic state, blood glucose and intramuscular lipid stores were measured 10 min after the termination of exercise. Glycemia was 38% lower in exercising compared with sedentary mice (Fig. 1*B*). Intramyocellular lipid (IMCL) droplets in the soleus were less abundant and of reduced size in running mice (Fig. 1, *C* and *D*). Thus a single bout of voluntary exercise effectively increased metabolic demand within skeletal muscle, shifting the metabolic state towards undersupply.

#### Mitochondrial membrane interactions.

SS mitochondria are clustered beneath the plasma membrane whereas IMF mitochondria are layered in an interconnected reticulum near Z-lines between sarcomeres (Fig. 2, *A* and *B*). Examination of mitochondrial morphology in both longitudinal and cross-sectional (transverse) planes can be achieved by mounting two different muscle specimens in orthogonal orientation for EM (Fig. 2*C*), or by optical sectioning of muscle fibers in different orientation by confocal microscopy (Fig. 2, *D*–*F*). The typical pair of seemingly spherical mitochondria observed in longitudinal sections (Fig. 2*G*) are in fact tubular and branched organelles when visualized in cross section (Fig. 2*H*; see also Ref. 29).

**Fig. 3.**
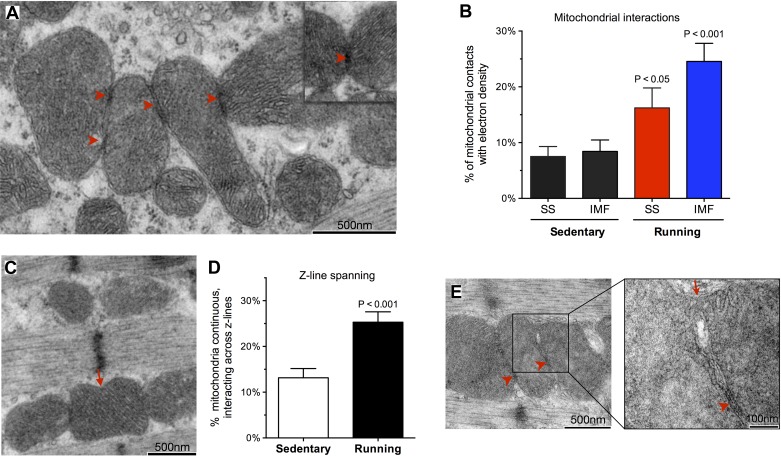
Increased mitochondrial membrane interactions following exercise. *A*: representative TEM images of soleus SS mitochondria linked by electron-dense contact sites (EDCS). *B*: proportion of mitochondrial contacts exhibiting an enhanced electron density in SS and IMF mitochondria, from sedentary and running mice. *C*: pairwise (top) and continuous (bottom, arrow) mitochondria across a Z-line. *D*: proportion of continuous or interacting IMF mitochondria across Z-lines. *E*: neighboring mitochondria tethered by EDCS (arrowheads) and connected by a narrow (≈50 nm) tunnel of matrix space. *n* = 14–16 myofibers analyzed per group.

Mitochondrial dynamics involve the direct contact between outer mitochondrial membranes of adjacent organelles, followed by rapid (within seconds) sequential fusion of the outer and inner membranes (49). Electron-dense structures physically linking outer membranes are recognized as EDCS upon examination of skeletal muscle mitochondrial with electron microscopy (Fig. 3*A*) (2, 43). The abundance of EDCS increased by 1.2 fold (*P* < 0.05) in SS, and by 1.9 fold (*P* < 0.01) in IMF mitochondria after exercise, indicating increased mitochondrial interactions (Fig. 3*B*). In addition, the proportion of IMF mitochondria interacting or physically spanning the Z-line increased by 93% (Fig. 3, *C* and *D*). Muscles of exercising mice also contained rare events of membrane-bound bridges connecting the matrix space of tethered mitochondria (Fig. 3*E*), consistent with processes of mitochondrial dynamics.

#### Mitochondrial morphology.

**Fig. 4.**
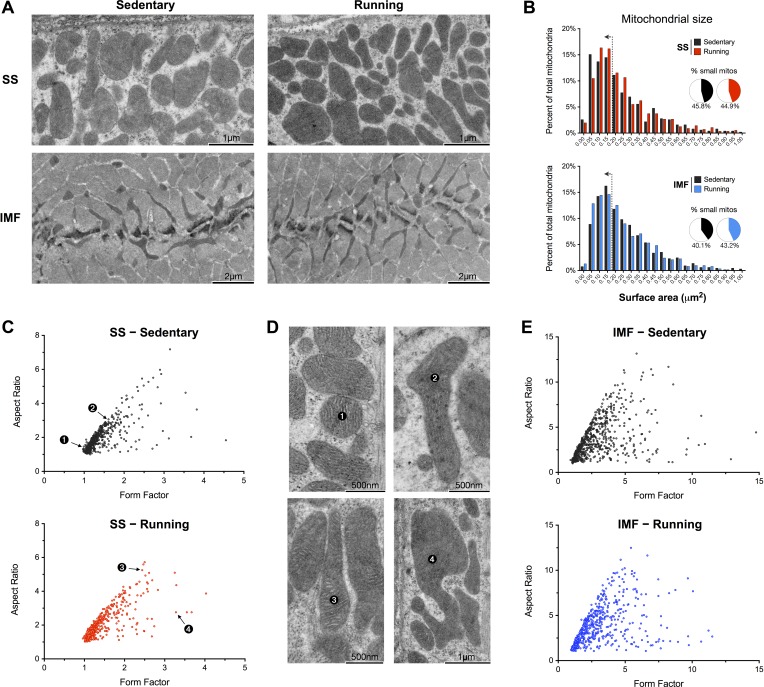
Mitochondrial size and morphology in sedentary and running mice. *A*: representative TEM micrographs of SS and IMF mitochondria from the soleus of sedentary and running mice. *B*: quantification of mitochondrial size shown as frequency distributions, for which the bin center is indicated. Inset shows the proportion of small mitochondria (<0.175 μm^2^; left of the dotted line on histograms). *C*: form factor and aspect ratio distributions for individual, manually-traced SS mitochondria. Numbered data points represent individual mitochondria shown in *D*. *E*: data as in *C* for IMF mitochondria. *n* = 505–653 mitochondria analyzed per group.

**Fig. 5.**
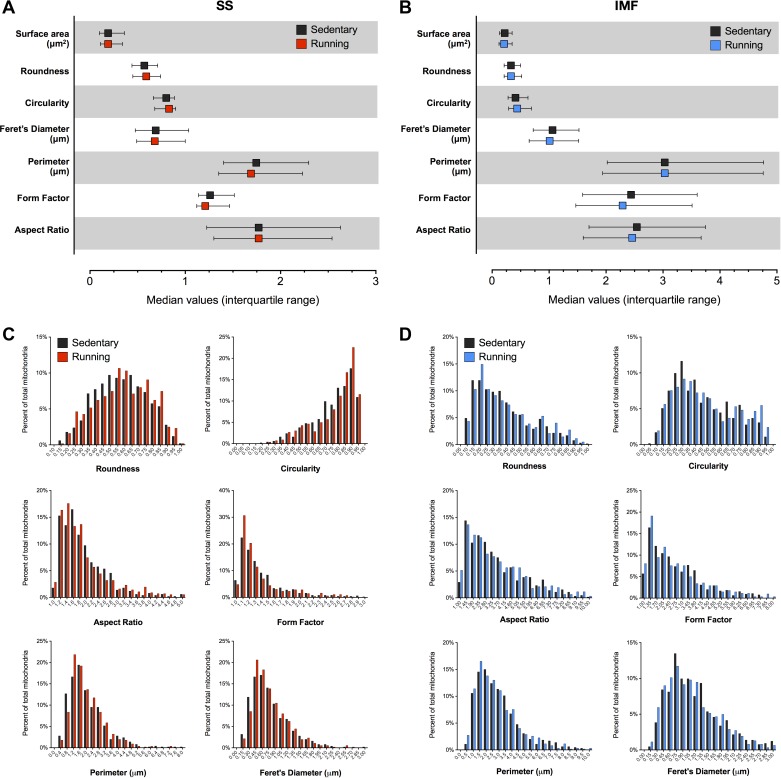
Mitochondrial morphology in sedentary and running soleus. Mitochondrial shape descriptors for SS (*A*) and IMF mitochondria (*B*). All nonsignificant vs. sedentary. *C* and *D*: frequency distributions for the same morphological parameters as in *A* and *B*.

Acute voluntary exercise did not alter mitochondrial ultrastructure (Fig. 4*A*). The fusion of neighbouring mitochondria is expected to *1*) increase mitochondrial size, and/or *2*) affect mitochondrial shape descriptors such as length (aspect ratio) and branching complexity (form factor). Mitochondrial size and morphology were quantified by manually tracing mitochondria from electron micrographs in both the longitudinal (for SS) and transverse/cross-sectional (for IMF) orientations. Exercise running did not affect mitochondrial size (Fig. 4*B*). Likewise, neither mitochondrial aspect ratio nor form factor were altered after exercise (Fig. 4, *C*–*E*). Other morphological parameters including roundness, circularity, perimeter, and Feret's diameter did not differ between sedentary and running conditions (Fig. 5, *A*–*D*).

#### Mitochondrial pro-fusion proteins.

**Fig. 6.**
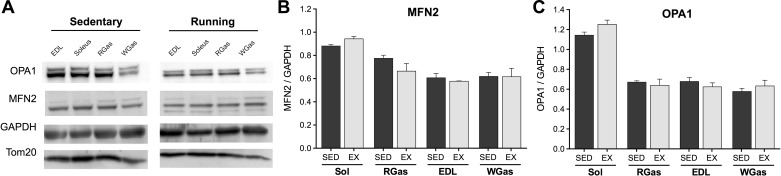
Mitochondrial fusion proteins in skeletal muscle of sedentary and exercised mice. *A*: Representative immunoblots for the mitochondrial pro-fusion proteins optic atrophy 1 (OPA1) and mitofusin 2 (Mfn2), the cytoplasmic marker GAPDH, and mitochondrial marker TOM20, in different muscles of the leg: extensor digitorum longus (EDL), the soleus (Sol), the red gastrocnemius (RGas), and the white gastrocnemius (WGas). Average protein abundance of Mfn2 (B) and OPA1 (C) normalized to GAPDH in different hind limb muscles. Data are means ± SE; *n* = 3 per group.

To determine whether the increased membrane interactions in exercised muscles could be related to changes in the mitochondrial pro-fusion proteins, we analyzed the canonical fusion proteins Mfn2 and OPA1 (Fig. 6*A*). The abundance of the outer mitochondrial membrane fusion protein Mfn2 (Fig. 6*B*) and the inner mitochondrial membrane OPA1 (Fig. 6*C*) were similar between sedentary and exercised animals.

## DISCUSSION

The ability of mitochondria to interact and fuse in skeletal muscle fibers is a relatively recently discovered property, and whether these processes are altered by exercise is unknown. To isolate the acute effect of exercise on mitochondrial dynamics, we applied a “nonstressful” voluntary running paradigm in mice, following their normal physical activity pattern, and quantified mitochondrial morphology on electron micrographs from the longitudinal and cross-sectional axes of skeletal muscle. Although neither mitochondrial size nor morphology were altered immediately after exercise in the soleus, mitochondrial membrane interactions were significantly increased, particularly in the IMF compartment where energy demand and mechanical stress might be greatest.

Based on in vitro studies indicating that energy deprivation can induce mitochondrial fusion and elongation (22, 46), our initial hypothesis was that the state of metabolic undersupply incurred during exercise—increased energy demand relative to supply—would provoke the elongation and enlargement of mitochondria. In addition, since oxidized glutathione (GSSG) can stimulate mitochondrial fusion (47), and skeletal muscle contraction can increase ROS production (44), this could further have stimulated mitochondrial fusion. However, excess ROS production triggered during exhaustive exercise (11) has been shown to trigger mitochondrial fragmentation in myotubes (15). Opposing forces promoting mitochondrial fusion on the one hand and fragmentation on the other may be simultaneously acting in the exercising skeletal muscle, possibly explaining the absence of overt morphological changes during or immediately after exercise.

Mitochondrial dynamics in skeletal muscle fibers may serve different potential functional roles contributing to normal mitochondrial function and skeletal muscle physiology (40). Physical tethers between mitochondrial membranes could *1*) promote the exchange of molecules (ions, lipids, proteins) between adjacent organelles (8, 48), which may help to coordinate the functioning of otherwise physically distinct mitochondria; *2*) protect healthy mitochondria from autophagy (22, 46) without necessarily engaging energy-consuming processes of complete outer mitochondrial membranes (OMM) and inner mitochondrial membranes (IMM) fusion; and *3*) serve as prefusion events by tethering organelles' outer membranes to facilitate their rapprochement and possible fusion upon the appropriate microenvironment signals. These speculative functions deserve further experimental clarification.

In mammalian cells, mitochondrial morphology is remodelled by a group of GTPase proteins, including mitofusins (Mfn1, Mfn2) and OPA1, located respectively within the OMM and IMM (52). Mitochondrial fusion involves the rapprochement of OMMs from adjacent organelles, followed by sequential fusion of the OMM and IMM (33, 49). Mitochondrial membrane interactions are therefore a prerequisite for mitochondrial morphology transitions. The short exercise duration and absence of recovery period may explain the absence of difference in the abundance of Mfn2 and OPA1 with acute exercise. A study in humans also reported no change in the abundance of Mfn1 or Mfn2 following a single bout of exercise (38). In contrast, the transcriptional regulation of mitochondrial fusion and fission proteins appears more rapidly regulated and correlates with functional exercise capacity (19). One study in rats reported the transcriptional upregulation of fission protein Fis1 *during* intense exercise, with a concomitant downregulation of the fusion proteins Mfn1 and Mfn2 (12). In the recovery phase (0–2 h after exercise), this was followed by an early upregulation of Mfn1, and a delayed upregulation of Mfn2 at the mRNA level (12). Other studies in humans (5, 24) and rats (27) have also found increased transcript levels for mitochondrial fusion proteins in response to chronic muscle stimulation or endurance exercise. However, transcript levels for those genes are likely to bear more significant effects on the long-term adaptations than on immediate changes in mitochondrial morphology. In addition, transcript and protein levels of the key players in mitochondrial dynamics may not directly translate into altered dynamics since the pro-fusion activity of Mfn2 and OPA1 are regulated by posttranslational modifications and processing (13, 45, 47), respectively. Finally, it is also possible that mitochondrial tethers are mediated by proteins or membrane properties that do not involve the canonical mitochondrial fusion machinery.

SS mitochondria are located beneath the plasma membrane, whereas mitochondria in the IMF compartment are positioned between myofibrils in proximity to the T-tubules and sarcoplasmic reticulum where most of the ATP is hydrolyzed during and between contractions. Functionally, differences exist between SS and IMF mitochondria (10, 16, 31), and their basal morphologically also differ greatly (see Fig. 4 and Ref. 43). Exercise could therefore differentially affect mitochondrial membrane dynamics in the SS and IMF compartment. Our results indicate that although the proportion of EDCS was equivalent in sedentary conditions (about 7–8% of contacts are electron-dense), the increase with exercise was more robust in the IMF compartment. Another notable finding was that IMF mitochondria spanning the Z-lines were nearly twice as prevalent after acute exercise. Because mitochondria form reticular networks in the transverse plane (along sarcomeric planes) of myofibers, it is hypothesized that interconnecting these planes *across* sarcomeres would enable the transfer of metabolites, ions, or membrane potential, thus contributing to coordinate mitochondrial function along the myofiber.

In conclusion, we show that mitochondrial morphology is not affected immediately after exercise, but rather that EDCS tethers identified by electron microscopy are increased. This represents a novel immediate adaptation of skeletal muscle, especially among IMF mitochondria. Defining the impact of fluctuations in energy metabolism on mitochondrial dynamics, both during exercise and sedentary behaviour, has important implications for deciphering the mechanisms responsible for the health effects of physical in/activity (4, 42). Remaining questions include the molecular nature of EDCS, their exact role, and their long-term effects on skeletal muscle physiology. The exercise duration and intensity required for their formation and dissolution, and whether they eventually translate into membrane fusion, elongation, and enlargement of mitochondria remains to be explored.

## GRANTS

M.P. was supported by a Canada Graduate Scholarship and a Michael Smith Foreign Study Supplement from the National Science and Engineering Research Council of Canada (NSERC), a Journal of Cell Science Travelling Fellowship, and holds a Canadian Institute of Health Research (CIHR) Postdoctoral Fellowship from the Institute of Neurosciences, Mental Health and Addiction as part of the Canadian Epigenetics, Environment and Health Research Consortium. Part of this work was performed in the Newcastle University Centre for Brain Ageing and Vitality supported by grants from the Biotechnology and Biological Sciences Research Council (BBRC), Engineering and Physical Sciences Research Council (EPSRC), Economic and Social Research Council (ESRC), Medical Research Council (MRC), the Wellcome Trust Centre for Mitochondrial Research and National Institute for Health Research Newcastle Biomedical Research Centre based at Newcastle upon Tyne Hospitals National Health Service Foundation Trust, and Newcastle University to D.M.T. Additional support came from NIH grant NS21328 and CA143351 and Simons Foundation grant 205844 awarded to D.C.W. Part of this work was supported by the Canadian Institutes of Health Research grant 86725 to B.J.G.

## DISCLOSURES

No conflicts of interest, financial or otherwise, are declared by the author(s).

## AUTHOR CONTRIBUTIONS

Author contributions: M.P., S.E.G., and D.M.T. conception and design of research; M.P., M.J.M., K.W., and K.S.L. performed experiments; M.P. and B.J.G. analyzed data; M.P. and D.M.T. interpreted results of experiments; M.P. and B.J.G. prepared figures; M.P. drafted manuscript; M.P., B.J.G., M.J.M., and D.M.T. edited and revised manuscript; M.P., B.J.G., M.J.M., K.W., K.S.L., S.E.G., D.C.W., and D.M.T. approved final version of manuscript.
